# Innovative Technologies to Improve Occupational Safety in Mining and Construction Industries—Part I

**DOI:** 10.3390/s25165201

**Published:** 2025-08-21

**Authors:** Paweł Bęś, Paweł Strzałkowski, Justyna Górniak-Zimroz, Mariusz Szóstak, Mateusz Janiszewski

**Affiliations:** 1 Department of Mining, Faculty of Geoengineering, Mining and Geology, Wroclaw University of Science and Technology, 50-370 Wroclaw, Poland; pawel.strzalkowski@pwr.edu.pl; 2 Department of Geodesy and Geoinformatics, Faculty of Geoengineering, Mining and Geology, Wroclaw University of Science and Technology, 50-370 Wroclaw, Poland; justyna.gorniak-zimroz@pwr.edu.pl; 3 Department of General Construction, Faculty of Civil Engineering, Wroclaw University of Science and Technology, 50-370 Wroclaw, Poland; mariusz.szostak@pwr.edu.pl; 4 Department of Civil Engineering, School of Engineering, Aalto University, P.O. Box 12100, FI-00076 Aalto, Finland; mateusz.janiszewski@aalto.fi

**Keywords:** innovative technologies, occupational safety, mining, construction

## Abstract

Innovative technologies have been helping to improve comfort and safety at work in high-risk sectors for years. The study analysed the impact, along with an assessment of potential implementations (opportunities and limitations) of innovative technological solutions for improving occupational safety in two selected sectors of the economy: mining and construction. The technologies evaluated included unmanned aerial vehicles and inspection robots, the Internet of Things and sensors, artificial intelligence, virtual and augmented reality, innovative individual and collective protective equipment, and exoskeletons. Due to the extensive nature of the obtained materials, the research description has been divided into two articles (Part I and Part II). This article presents the first three technologies. After the scientific literature from the Scopus database was analysed, some research gaps that need to be filled were identified. In addition to the obvious benefits of increased occupational safety for workers, innovative technological solutions also offer employers several economic advantages that affect the industry’s sustainability. Innovative technologies are playing an increasingly important role in improving safety in mining and construction. However, further integration and overcoming implementation barriers, such as the need for changes in education, are needed to realise their full potential.

## 1. Introduction

Modern mining and construction are industries that prioritise efficiency, productivity, and most importantly, worker safety. Both industries are characterised by dynamic work environments, employee exposure to numerous hazards and highly complex technological processes. Effective safety management in these sectors, therefore, requires strict compliance with regulations and standards, as well as the implementation of innovative technological solutions. Therefore, technological development plays a key role in minimising risks and protecting employee health [1,2,3,4].

Technological advances over the past two decades have led to the rapid development of tools that support the monitoring, analysis, and minimisation of workplace hazards. Industry 4.0 technologies are increasingly being applied in industrial environments. Their potential to improve occupational safety in mining and construction is the focus of an increasing amount of scientific research and industrial implementation [5,6,7]. Thanks to innovations such as real-time monitoring systems for working conditions, advanced automation, robotics, and artificial intelligence, risks can be detected at an early stage and rapid preventive action taken. Smart personal protective equipment and digital solutions to support employee training are also becoming more popular [3,4,8,9,10].

In the mining sector, where conditions are often characterised by limited visibility, high levels of dust, extreme temperatures, and a constant risk of natural disasters such as sinkholes, methane explosions, and rockbursts, the use of modern technology is essential for early warning and prevention systems. Integrated sensor systems with wireless networks enable continuous monitoring of environmental parameters and machinery condition, while artificial intelligence algorithms can predict hazardous situations based on historical and current data [11]. In construction, however, where work is carried out in various field conditions under time pressure with numerous subcontractors, managing risks through digitising construction processes, automating tasks, and using intelligent systems to control access and locate workers is becoming important [12].

In both industries, technologies that support employee training and development are also becoming increasingly important [3,13,14,15,16]. Virtual reality (VR) simulators, digital models of facilities and interactive instruction manuals enable personnel to be effectively prepared to work in harsh environments without exposing them to real danger during the training process. Innovative technologies also support audit and safety inspection processes—for example, unmanned aerial vehicles, 3D scanners, and mobile inspection applications enable a quick and precise assessment of the condition of facilities and infrastructure, thereby minimising the need for dangerous human activities.

**Figure 1:**
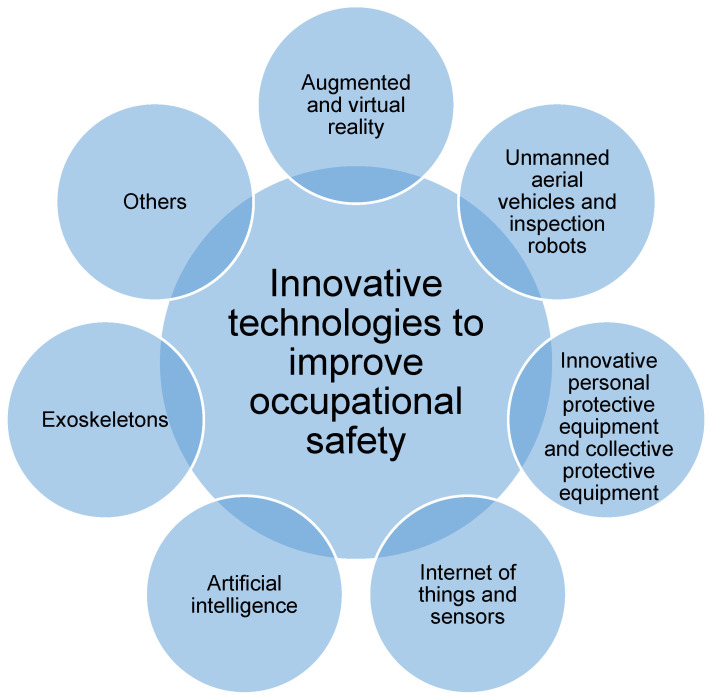
Innovative technologies to improve occupational safety in mining and construction industries (own study).

Modern technologies play a vital role in enhancing occupational safety within the mining and construction industries. Innovative solutions minimise the risk of accidents while increasing operational efficiency and improving working conditions. It is worth mentioning that current technological developments allow processes and tools to be optimised not only by industry but also according to the size of the plant or enterprise, and the gender and age of the workers in the sector. Figure 1 presents the most important solutions that realistically affect the level of occupational safety in these sectors.

This article aims to analyse and systematise the most important modern technologies currently in use (or potentially to be implemented) in the mining and construction sector, considering their impact on improving occupational safety. We analysed solutions that are already functioning as independent techniques, as well as the possibility of combining them. Particular emphasis was placed on identifying technological developments that could reduce occupational accidents, improve the efficiency of occupational safety management, and enhance the quality of training and supervision of compliance with occupational safety and health (OSH) procedures. The analyses conducted will provide insight into the barriers to implementing modern technologies and define recommendations for further research and technological development to improve working conditions and protect the lives and health of workers in the two analysed sectors.

Due to the extensive range of technologies used to improve occupational safety and their wide application, the discussion is divided into two parts. This paper, which is Part I of a series, analyses and describes smart technology-based solutions using sensors and/or advanced information technologies, such as unmanned aerial vehicles, inspection robots, the Internet of Things and sensors, and artificial intelligence. Part II will discuss technologies that are used directly by humans, such as augmented and virtual reality, personal and collective protective equipment, and exoskeletons. Section 4 presents a comparative table of all the modern technologies selected for the study. This allows the combination of the technologies discussed in this section with all other technologies to be discussed, including those in Part II.

## 2. Materials and Methods

**Figure 2:**
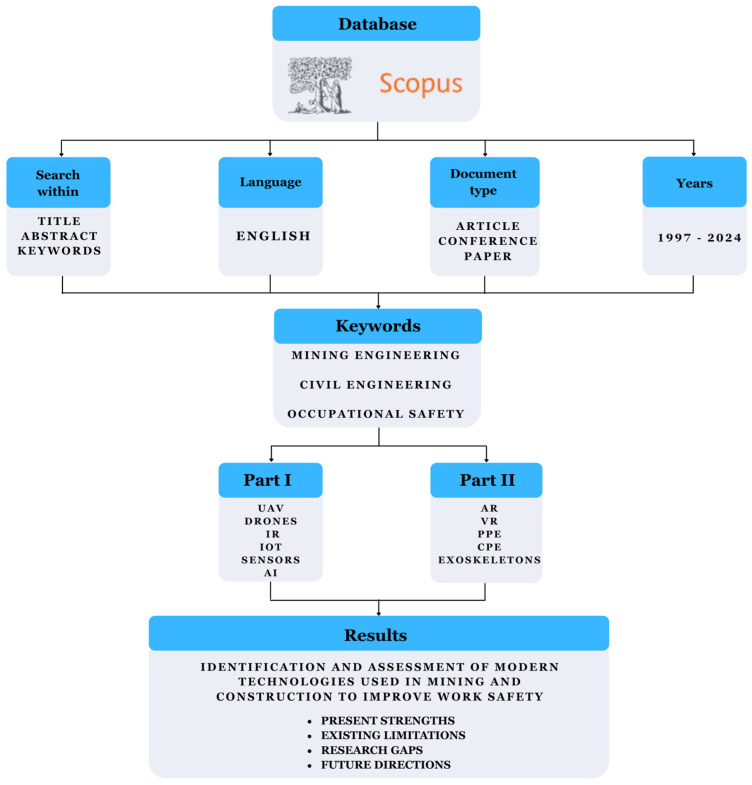
Diagram of the methodology of the conducted research (own study).

The literature review employed a desk research methodology, whereby research is conducted from behind a desk. Also known as secondary analysis, it involves collecting, analysing, and interpreting published data. In the context of a literature review, desk research relies on foundational sources such as academic articles, reports, books, statistical data, and publications from research institutions. This method enables the efficient and rapid acquisition of information, eliminating the need for field research [17,18]. A key element of desk research is critically analysing and evaluating the reliability, validity, and relevance of sources to the research topic. Despite the fact that desk research allows specific research problems to be answered with little financial and organisational effort, it is still an underutilised analytical technique in research [19,20]. To illustrate the methodology undertaken and the subsequent steps of conducting research on the issue, the following diagram is presented (Figure 2). The same research methodology was used in both parts of the articles (Part I and Part II). The only difference in the methodology is the keywords used in the Scopus search.

**Figure 3:**
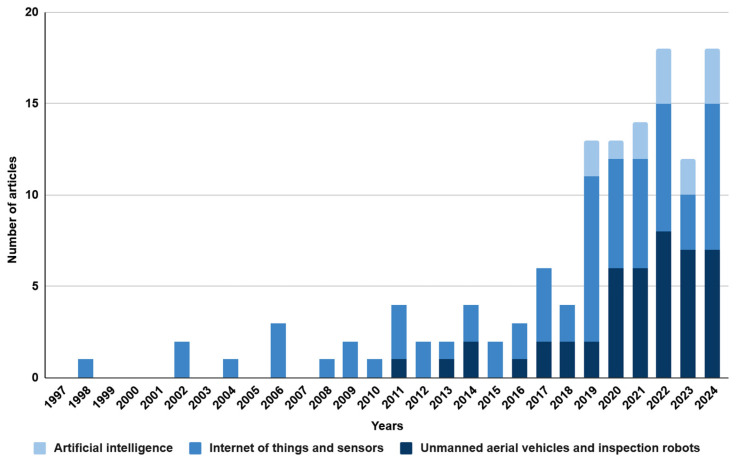
The number of literature results for a given technology in mining engineering in specific years (own study based on Scopus database).

**Figure 4:**
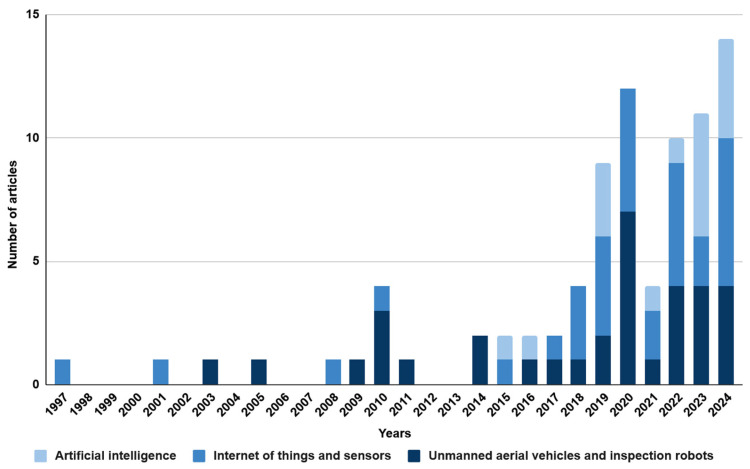
The number of literature results for a given technology in civil engineering in specific years (own study based on Scopus database).

Source analysis was carried out based on the keywords established in Table 1. Two sets of keywords were selected: ‘mining engineering’ and ‘civil engineering’. The research was conducted using the Scopus database. The literature review covered the period from 1997 to 2024 and included only original scientific articles and conference papers written in English. As a result, a total of 209 records were obtained. All the selected publications were analysed in detail, and those not related to occupational safety in mining and civil engineering were excluded. Based on these publications, the technologies used to improve occupational safety were characterised. The analysis revealed an increasing interest in the topic, which may suggest the development and popularity of innovative technologies and their application in various areas of mining and civil engineering. The above conclusion is illustrated in the graphs (Figure 3 and Figure 4). The only difference in the methodology is the keywords used in the Scopus search.

A review of the literature was conducted to assess the potential for combining technologies to improve occupational safety. Areas for combining technologies were proposed, as were the limitations of inter-technological integration.

## 3. Results

### 3.1. Unmanned Aerial Vehicles and Inspection Robots

Technological advances are increasingly supporting occupational health and safety in high-risk sectors such as mining and civil engineering. One notable innovation is the use of unmanned aerial vehicles and inspection robots. These remotely controlled or autonomous devices can collect high-resolution data, monitor work processes and environments, and carry out tasks in areas that are too dangerous or inaccessible for humans. Interest in these devices has grown significantly over the past three decades. Although initially developed for military purposes, they are now widely used in industry for infrastructure development, process evaluation, environmental monitoring, and workplace safety. In the context of occupational safety, these devices offer numerous advantages. They facilitate real-time inspections and the monitoring of working conditions, as well as supporting rescue operations, all without endangering workers. This is particularly important in mining and construction, where physical risks and changing working conditions are commonplace. Equipped with sensors, vision systems and software, these devices can interact with their environment and collect data about the objects being inspected. Often, these robots are unmanned aerial vehicles (UAVs).

#### 3.1.1. Mining Engineering

In the mining industry, inspection robots are mainly used to monitor production processes to ensure work safety [21,22,23,24,25,26], and for the planning and execution of rescue missions, mainly in underground mines equipped with laser scanners (LIDAR) and thermal imaging cameras [27,28]. Ruiz-del-Solar et al. [25] describe the process of the University of Chile’s Centre for Advanced Mining Technology struggling with low research funds, low trust from local mining companies, and the government developing its activities for the mining industry since 2008. The centre has developed various technologies, including an autonomous vehicle for mining environments without global positioning system coverage, an inspection UAV equipped with a thermal imaging camera, and a vision system for remotely operated vehicles in adverse climatic conditions. In the second decade of the nineteenth century, the Council for Scientific and Industrial Research (CSIR) decided to improve occupational safety in the South African mining industry by establishing The Centre for Mining Innovation (CMI), conducting research that includes the testing and validation of inspection robots equipped with sensors to monitor the mine environment [29,30].

The use of explosives is one of the most dangerous activities in open-pit mining, involving hazards such as scattering of rock fragments (flyrock), overpressure, and ground vibration. UAVs have proven useful in improving blast design and reducing hazards by providing pre- and post-blast data from a safe distance. Nguyen et al. [31] have demonstrated the value of UAV-based predictive modelling for rock debris scattering. This has significantly improved safety by enabling the earlier implementation of preventive measures and the more accurate prediction of hazards. The authors developed a predictive model for flyrock using UAV-acquired terrain imagery in conjunction with a neuro-fuzzy inference system (ANFIS). Their approach improves blast risk zoning and helps prevent injuries from uncontrolled debris scattering. UAVs were used to acquire high-resolution models of the ground surface, which then served as input for the predictive system. This study shows that UAVs can significantly improve safety during blasting operations by reducing uncertainty in hazard prediction and minimising human presence in high-risk areas.

UAVs are also widely used for monitoring geological structures, deformation patterns, and field hazards. Identifying these factors is critical to mine safety, as failure to do so can lead to landslides, rockfalls, or structural collapses. Studies by Layek et al. [32] and Cao et al. [33] examined the use of UAVs for monitoring slope stability and highlighted the significant challenges involved. Their findings confirm the effectiveness of UAVs in providing accurate and timely data, which is crucial for preventing landslides and protecting workers.

Another application of UAVs in improving safety in mining is environmental monitoring. Luo et al. [34], Banerjee and Raval [35], and Rysbekov et al. [36] have emphasised the potential of UAVs for geophysical exploration and sustainable mining practices, indicating important safety implications through more effective environmental monitoring and hazard identification. Hatfield et al. [37] and Xiao et al. [38] detailed how UAV systems can be used to manage environmental hazards and ensure operational safety in open-pit mines. They emphasised that real-time data collection and hazard identification are major safety benefits. Similarly, Alias and Udin [39] explored how UAVs could support occupational safety by improving situational awareness and enabling a faster response to emergencies.

Several studies, including those of Yao et al. [40] and Li et al. [41], have demonstrated advanced applications of UAV, such as automatic defect detection in mine infrastructure, significantly reducing workers’ exposure to hazardous environments. Integrating digital twin and machine learning technologies into UAV systems further enhances predictive capabilities, as demonstrated by Qiao [42] and Somanagoudar and Mérida [43], pointing to promising directions for future research and implementation.

#### 3.1.2. Civil Engineering

A previous works [44,45] reviewed and analysed modern technologies used in the construction industry to improve structural safety throughout the life cycle of a construction project. This research identified a set of technologies based on robotics, automation, BIM, VR, AR, AI, and IoT technologies. The study found a clear link between the use of new technologies and the safety performance of building structures.

The main cause of fatal accidents in construction is falls and slips resulting from various environmental hazards, such as the following: slippery surfaces and exposed edges, for example. In order to reduce the risk of the aforementioned accidents, it is crucial to locate potential fall hazards. In their studies [46,47], the authors replaced conventional construction safety monitoring methods with a multiagent robotic system that automatically detects and locates potential hazards in construction. This study focused mainly on fall hazards of the same level. The proposed robots detect and locate hazards by integrating simultaneous locating and mapping, along with planning safe paths. The proposed system can make a significant contribution to safety management at work in the construction industry. Another example of a mobile robot application for worker safety surveillance accepted by the Occupational Safety and Health Administration (OSHA) is a robot (simultaneous localisation and mapping) based on a complex deep learning module using neural networks and SLAM. This module detects a construction worker’s failure to wear a helmet under various conditions on a construction site and facilitates the control and supervision of his safety [48].

Sanson [49] and Górski et al. [50] investigated the use of inspection robots in the form of UAVs in the construction industry, highlighting the improvement of workplace safety through real-time data collection from the construction site and monitoring of structural health. Their research indicates that UAVs have great potential to reduce human exposure to unsafe working conditions. Franco et al. [51] highlighted the role of UAVs in promoting sustainability and safety through detailed monitoring of construction sites, identifying hazards, and documenting compliance with health and safety regulations. Similarly, Bae et al. [52] described the SMART SKY EYE system, which uses UAVs for initial construction safety assessments, significantly increasing the ability to detect potential hazards early on in construction projects. Guptaa and Nairb [53] and Liang et al. [54] focused on the challenges and innovative solutions involved in inspecting critical infrastructure, such as bridges, using UAVs. They emphasised the effectiveness of this technology in the early detection of defects and risk mitigation.

Another safety application of UAVs on construction sites is monitoring worker safety. Pena et al. [55] presented a novel UAV designed to monitor workers’ health in real time. Francesco et al. [56] described how UAVs can be used in rescue operations, particularly after earthquakes and other large-scale disasters. This significantly increases the safety and efficiency of rescue operations.

Recent developments include the integration of artificial intelligence and machine learning into UAV systems, as demonstrated by Hariri-Ardebili et al. [57] and Chen et al. [58]. These technologies significantly enhance the capabilities of UAVs for autonomous navigation, object tracking and real-time threat detection, pointing to promising directions for future research and practical implementations. The growing use of UAVs is also finding a place in construction education and training, as highlighted by Harper et al. [59], demonstrating their effectiveness in teaching health and safety principles and increasing hazard awareness among construction professionals.

### 3.2. Internet of Things and Sensors

The Internet of Things (IoT) can be defined as a network of objects and devices that connect to each other via the Internet using sensors, interfaces and software. This technology can, therefore, be used in various scientific fields to monitor the status and safety of people, devices, and systems. The IoT is revolutionising the approach to occupational safety in demanding industries such as mining and construction. Smart sensors and wearables monitor key environmental parameters such as dangerous gas concentrations in mines and noise and vibration levels on construction sites, enabling the early detection of hazards. Real-time, IoT-based location systems enable the tracking of workers’ locations, which is crucial for emergency situations and managing teams over wide areas. Smart helmets and workwear equipped with sensors can monitor workers’ physiological parameters, such as heart rate and body temperature. This alerts them to potential fatigue or deterioration. The analysis of data collected via IoT devices provides valuable insights into potential risks and unsafe behaviour patterns, enabling the proactive implementation of preventive measures and the optimisation of safety procedures. In mining, the IoT can be used to monitor pit stability and machine condition, predict failures, and minimise the risk of accidents. In construction, unmanned aerial vehicles equipped with sensors and cameras and connected to IoT systems can inspect hard-to-reach areas and identify potential hazards or violations of health and safety regulations. Integrating IoT with alarm and communication systems improves emergency responses by enabling rapid notification of relevant services and coordination of rescue operations. This significantly improves the safety and health protection of workers in these key economic sectors. Using modern technologies such as sensors or sensor systems, which are often based on the IoT, in the mining and construction industries can lead to increased productivity, reduced costs, improved occupational safety and operational efficiency in production and execution processes, more reliable acquired data and information, and an improved quality of the environment [60].

#### 3.2.1. Mining Engineering

The use of wireless sensor networks to regularly monitor mining activities, safety conditions, and the health of miners working underground is a frequently discussed research topic in the literature [61]. The results of the literature search were categorised into three main categories: monitoring and tracking the location of employees, machinery, and equipment; monitoring the physiology and kinematics of miners’ bodies; and monitoring the working environment.

The first category includes sensors that monitor the location of machinery, devices, and employees. These sensors are usually wireless and detect obstacles near mining machinery, warning the operator of hazards such as other machines, workers, embankments, or the walls of an excavation [62,63,64,65,66]. Sensors are also used on machines to alert workers to machinery operating in their vicinity [67,68,69]. Proximity sensors are also used in workers’ clothing as personal alarm devices (PADs) to accurately determine the workers’ position in relation to mining machinery [70] and in workers’ helmets [71]. Wearable sensors use an intelligent approach with warning systems equipped with image recognition, motion detectors, and AI. The use of this proximity detection technology has increased operator safety and reduced the incidence of serious fatal accidents.

The second category of sensors includes non-invasive wearable sensors that monitor the physiological activities of miners working in extreme conditions that pose a threat to their health and life [72]. The paper [73] presents research on a device (a T-shirt as the first layer of protective clothing) for the continuous monitoring of the measurement of physiological variables of miners (electrocardiogram, respiratory activity, and body temperature) and environmental variables (ambient temperature and relative humidity) working at high altitudes (>2000 m above sea level).

Most research focuses on developing sensors in the third category, which monitor the condition of the working environment. These sensors measure parameters such as humidity, temperature, dust and gas pollution, noise and machine vibration, which affect occupational health and safety. This group includes sensors that measure the temperature of machines, devices, and technical infrastructure [74]. Increased temperature can damage machinery and equipment, contributing to the risk of fire and electric shock. Therefore, current sensors are used in mines to protect employees from electric shock caused by damage to machinery, equipment, or technical infrastructure [75]. The National Institute for Occupational Safety and Health (NIOSH) conducts research in excavations using humidity and temperature sensors to assess the thermal environment of mines, which are treated as enclosed spaces where people may be confined and seek shelter during a fire [76]. In hard coal mines, workers are exposed to dust and gas hazards. The biggest of them is the threat of carbon monoxide, methane and coal dust, which can lead to the poisoning of employees or fire. Therefore, wireless sensors are installed in mines to detect carbon monoxide [77,78,79], methane [80], coal dust or other gaseous pollutants resulting from the operation of machinery and equipment [81]. If a fire has already occurred in a mine, fire monitoring sensors are used to provide technical support during fire area management and ensure work safety in the mine [79,82,83,84,85]. In underground mines of metal and non-metal ores, sensors are also used to detect airborne contaminants that threaten the health of miners. Examples of such sensors include, m.in, a smart system for monitoring and controlling air quality in a mine in real time with built-in countermeasures to reduce high concentrations of air pollutants (smart monitoring and control (SMAC)), developed by the Occupational Safety and Health’s Spokane Mining Research Division (SMRD) [86], and a prototype of an information management system in coal mines to detect hazards [87,88]. Another dust hazard is silica, which affects the health of employees of mines, cement plants, and processing plants. Consequently, research is being conducted to develop a sensor that can constantly monitor people working in dusty environments using a Sharp GP2Y1010AU0F optical dust detector and an RFID card reader [89], as well as a low-cost sensor that can monitor silica dust in real time [90]. Excessive noise and vibration from machinery also pose a significant threat to workers. Therefore, research is being conducted to develop sensors to measure noise in the work environment to determine its level and impact on the quality of work [91]. In underground mines, there are seismic hazards related to the operation of the rock mass. Therefore, to improve occupational safety, research is being conducted into the use of new sensors to measure the impact of seismic loading on soils [92]. Seismic sensors are also used in mines [93,94,95,96,97] to monitor the stability of underground structures (excavations and caverns storing natural gas and crude oil) and surface structures (foundations, rocks, and slopes).

The National Institute for Occupational Safety and Health (NIOSH), which is the federal institute responsible for conducting research and making recommendations for the prevention of work-related injuries and diseases, takes a comprehensive approach to research into the use of sensors in mining. An example of NIOSH’s comprehensive approach is the Gemini-Scout mine rescue robot developed together with Sandia, equipped with sensors that enable the identification of the environment and hazards at the site of an accident in a mine before rescue workers [98]. To monitor the condition of excavations in hard-to-reach and dangerous places, NIOSH employees have also developed a system that enables automated flights of unmanned aerial vehicles (UAVs) equipped with sensors (LiDAR, infrared and vision sensors). This allows the condition of machinery and equipment to be monitored, for example, as well as dangerous areas to be identified [99].

Sadeghi et al. [61] found that innovative technologies in the form of wireless sensor networks should be implemented in the mining industry. Further research should also be carried out on the development of systemic solutions for occupational health and safety management in underground mining, the analysis and management of big data, the deployment of monitoring systems based on wireless sensor networks, the integration of wireless sensor systems with other communication systems and their autonomy. Conducting research in these areas can help improve the efficiency and applicability of wireless sensor network technology for OHS in the mining industry in order to achieve a holistic OHS management system for underground miners using sensor networks to create healthier and safer mining workplaces in the near future.

With regard to applications in mining, two papers reviewed IoT-based technology for monitoring worker safety. Flores-Castañeda et al. [60] reviewed modern technologies used in the mining industry and assessed their environmental impact, as well as analysing the benefits of their use. This technology is based on creating a network through which various devices can communicate with each other. This enables remote monitoring and access to real-time information about the activities performed by mine employees. Sensors integrated into workwear can monitor employees’ health and physical conditions and thus assess urgent medical situations. Additionally, IoT can be used to monitor environmental conditions such as temperature, humidity, and air quality to identify hazardous situations. Therefore, IoT technology can improve efficiency, reduce human intervention, increase production, reduce pollution, and ensure occupational safety. Conversely, paper [61] reviewed the use of wireless sensors (wireless sensor networks, WSNs) to improve occupational safety in underground mines. The conclusion was that WSN technology should be adapted to IoT infrastructure, making it a reliable and promising approach to the regular monitoring of mining activities and the health and safety conditions of miners working underground. This will make it possible to use the technology for the real-time monitoring and tracking of mining assets (employees and equipment), improving transport safety, monitoring dead zones in the excavation, improving rescue operations, and monitoring miners’ health. A subsequent paper [100] presents a smart safety application for real-time safety monitoring and on-site operations management. This consists of a range of IoT devices and a central monitoring system. The app can be used for personal protective equipment and access monitoring, as well as for human fall detection, SOS activation, and emergency response. This system has been implemented in an oil processing plant, among other places.

The paper [87] presents work on an IoT-based integrated command centre comprising a mine map (AutoCAD), an air monitoring system (AMS), and the real-time location of miners. This centre enables real-time emergency response management in underground mining operations, thereby improving operational safety. Another example of IoT technology being applied in mining is the MineBot ground mobile robot, which was developed at the Mechatronics Laboratory at the Mechatronics Laboratory at Ricardo Palma University for studying and detecting chemical, physical, and biological agents in underground mines [101]. The next two publications describe research into using IoT-based technology in mining machinery to improve worker safety. Paper [67] describes the implementation of an IoT-based machine monitoring system by the National Institute for Occupational Safety and Health (NIOSH) at Central Pre-Mix. In contrast, a paper [102] describes research into using an IoT-based wireless COTS IoT system to improve safety for miners working on conveyor belts. This system consists of sensors and controllers that monitor the condition of the conveyor. The tests were carried out in laboratory conditions.

#### 3.2.2. Civil Engineering

Worldwide, the number of studies on the use of modern technologies to identify risk factors for construction workers is growing. In this context, wireless sensors are widely used to recognise various risk factors affecting construction workers’ safety, providing managers with a ‘smart eye’ to recognise harmful environments and workers’ biometric information and providing active personal protective equipment. They also support the development of technologies to minimise dangerous behaviours resulting from identified risk factors, as well as programmes for the dynamic assessment, analysis, and optimisation of employee decision-making processes [103].

Wearable sensors that collect physiological information about workers, monitor their movement and location, and monitor their environment to provide active protection against hazards, such as detecting hazardous materials like dust, noise, or gas leaks, are used to manage the safety, health, and well-being of workers in the construction industry [104].

The National Institute for Occupational Safety and Health (NIOSH) conducts research on the ergonomics of construction tasks with risk identification and subsequent risk reduction. Construction workers were equipped with two smartphones to collect data from gyroscopes, accelerometers, and linear accelerometers. This data was analysed to assess workers’ musculoskeletal disorders and design an ergonomic working environment [105]. Similar studies have been conducted in laboratory settings [106]. Another proposed sensor for studying the ergonomics of construction work is footwear insoles, which enable the quantitative determination of pressure on the soles while performing construction activities. The collected and analysed data allowed joint loads to be calculated and an accurate ergonomic assessment to be made in order to determine risk factors for MSDs [107]. Subsequent laboratory and field studies have developed a non-invasive, automated method of assessing physical fatigue in construction workers using deep learning algorithms, biomechanical analysis, and a construction worker physical fatigue model. This method can provide an objective assessment of fatigue at the joint level throughout the entire work process. It uses sensors that capture 3D motion from RGB camera images. A fatigue model is then used to calculate current joint capacity and provide a fatigue index based on joint function history. The proposed method has great potential for the ergonomic organisation of construction tasks [108]. To assess the risk to the musculoskeletal system of roofers’ knees, a sensor was designed to monitor their condition. This was tested in laboratory conditions while the workers were in a kneeling position on a sloping roof surface [109]. Another example of monitoring work to improve quality is the full-body fatigue monitoring and analysis system developed by [110], which is equipped with a critical power (CP) model and a bioenergetic model. It also uses a wrist-worn heart rate monitor.

In terms of occupational safety management in the construction industry, research is being conducted into the use of sensors to monitor the real-time location of employees (RTLS) and topography. These tests are carried out to identify hazards on construction sites. This knowledge enables management on construction sites to reorganise their work [111,112]. Employees are located using a helmet equipped with Nodemcu technology that transmits their real-time location via an Android smartphone. After a strong impact, the GPS Shield takes the coordinates of the accident and notifies the relevant emergency services. This system provides a rapid warning of accidents, thereby increasing safety in the construction industry [113].

An important element in construction is scaffoldings that serve as a temporary structural element to support workers, equipment, and materials during construction work. In previous research [114], a method was proposed for real-time modelling of scaffolding boundary conditions and design parameters by employing wireless strain gauges integrated with Arduino modules in an IoT-based system. This results in deformation data being obtained directly from the scaffolding structure, enabling the safety of the scaffolding to be monitored. Tests were also carried out on the scaffolding using an ambient intelligence (AmI) system consisting of a microcontroller, microwave sensors, a light-emitting diode (LED), and an audible alarm. This system enables construction workers’ safety to be monitored on site, thereby reducing the risk of falls from height [115].

In the construction industry, overhead power lines pose a significant risk to workers. If damaged, for example by mobile machinery, they can deliver an electric shock to operators. Current sensors are, therefore, used to warn workers before a machine comes into contact with an overhead line, thereby improving operator safety [75]. Another solution to protect workers from electric shock is an electric field sensor worn on the trouser leg, which activates a visual and audible alarm in case of danger [116].

The work by Tabatabaee et al. [117] was a literature review on the use of IoT-based technologies to manage occupational safety in the construction industry. The paper analyses technologies such as: RFID, ultrasonic detection and infrared access technologies, sensors, vision-based technologies, wireless communication, wearable technologies, MEMS, accelerometers, smart devices and watches, location sensors, ultra-wide band (UWB) technologies, Bluetooth, Zigbee, real-time feedback, GPS, lasers, GIS, gyroscope sensors, location tracking, biosensors, Wi-Fi modules, photoresistors, optical sensors, force stretchable resistors, touch sensors, BLE beacons, integrated physical and virtual technology, and augmented hearing protection technology. During the analysis of the above-mentioned technologies, 18 barriers were identified as hindering their implementation in construction projects in order to improve occupational safety. Also, in another study [118], the limitations of BIM and IoT technologies in improving building safety in developing countries were examined.

The study in [119] presents the design of a system aimed at monitoring the performance, health, and safety of construction company employees. This system is based on deep learning technology, the Industrial Internet of Things (IIoT), wireless access points, and smart bands, shoes, and helmets. The next two publications describe research into developing sensors to enhance occupational safety in the construction industry using IoT technology. Study [114] details the use of an Arduino module in wireless strain gauges to collect real-time deformation data from scaffolding used in construction. This data enabled the modelling of the boundary conditions and design parameters of the scaffolding structure according to its load conditions. This deformation data was sent to a finite element method (FEM) model to estimate the scaffold’s real-time structural behaviour. These studies were carried out to monitor the safety of structures and improve work safety in the construction sector by preventing the collapse of temporary structures. On the other hand, the paper [113] describes the use of a smart safety helmet equipped with IoT-based Nodemcu technology for rapid warning of accidents in the construction industry.

### 3.3. Artificial Intelligence

In recent years, artificial intelligence (AI) has been becoming one of the key tools in many industrial fields, and its application is opening up new opportunities for occupational safety, particularly in mining and construction. The nature of these industries is characterised by a distinct set of risks, necessitating the implementation of contemporary solutions that extend beyond mere monitoring of working conditions to enable a rapid response to potential hazards. The utilisation of artificial intelligence, with its capacity to process voluminous data sets, discern patterns, and formulate decisions in real time, is emerging as a pivotal component of strategies aimed at enhancing safety. Research on the use of AI in the context of occupational safety in mining and construction focuses on several key aspects: analysis of real-time data from sensors placed in different parts of mining plants or construction sites, analysis of behaviours and situations that may indicate potential dangers, support of processes related to prevention, prediction, on the basis of collected data, the moments when the risk of an accident is highest, allowing the implementation of appropriate preventive measures. In the context of the use of AI in monitoring the health of workers, research is required to develop systems that support the recognition of symptoms of fatigue, stress, or other factors that can affect the ability of workers to perform tasks safely. Furthermore, AI algorithms are being used to analyse accident data in order to identify the causes of accidents and develop more effective prevention methods.

#### 3.3.1. Mining Engineering

The utilisation of artificial intelligence in the context of mining operations holds considerable potential for enhancing occupational safety. This technological approach can play a pivotal role in the reduction in accidents and the enhancement of procedures for monitoring work processes. Mining, due to its specific and often dangerous working conditions, requires advanced technological solutions to predict hazards, automate technological processes, and improve response to dangerous situations.

The nature of employment within the mining industry is such that workers are exposed to a variety of natural, technological, and other hazards. In this context, artificial intelligence (AI) can be employed to analyse data collected via sensors and cameras deployed in mines, facilitating a more rapid detection of anomalies and the implementation of preventive measures. Zheyuan et al. [120] utilised safety robots that were based on a system of cameras and sensors, which were designed to collect data that described work processes. The subsequent processing of this data via artificial intelligence was undertaken to enhance the occupational risk assessment process, thereby facilitating more effective risk management. This enhancement was achieved by leveraging the data collected via safety robots. An alternative solution was proposed by Kim and Choi [71], who suggested the use of sensors and cameras installed on a helmet. The development of an intelligent system to issue warnings to workers regarding the proximity of objects to them was informed by the utilisation of imagery and artificial intelligence.

The application of artificial intelligence in the domain of mining has been instrumental in the monitoring of the health and safety of workers who are exposed to prolonged physical and mental stress, as well as physical and chemical agents. Contemporary health monitoring systems are predicated on the analysis of data from devices worn by workers, including armbands that monitor physical activity, heart rate, and exposure levels to noise, dust, and toxins. The utilisation of AI algorithms facilitates the continuous monitoring of health status, thereby enabling the early detection of health concerns, including but not limited to reduced fitness and fatigue, which can potentially result in accidents. Conversely, the utilisation of AI is a viable option when selecting measures to safeguard workers by substantiating their work environment. Imam et al. [121] proposed the use of AI to detect the correctness of personal protective equipment used in mining.

The utilisation of AI has been demonstrated to be a highly effective tool in the analysis of mining accidents. This analysis enables the identification of the underlying causes and the identification of the characteristic situations that lead to incidents. The utilisation of algorithms for the analysis of data pertaining to previous accidents, working conditions and actions taken in real time facilitates risk modelling and the development of more effective prevention strategies [122,123]. The capacity of AI algorithms to analyse extensive data sets, including accident reports, machine condition data, and meteorological data, facilitates the identification of potential risks and the proposal of procedural modifications.

The use of AI has also been demonstrated to be effective in the context of autonomous mining machines [124,125]. The utilisation of artificial intelligence has enabled these machines to execute complex tasks autonomously, thereby minimising the risks associated with human involvement in high-risk processes and facilitating the identification of hazards. Automated transportation systems can move raw materials more efficiently and safely, avoiding the risk of accidents caused by human error.

#### 3.3.2. Civil Engineering

Technological advances, particularly the development of artificial intelligence (AI), are creating new opportunities to enhance safety in the construction industry. The capacity of artificial intelligence to predict risks, automate risk analysis, and monitor working conditions in real time is considerable.

One of the main areas of AI use in construction is risk prediction and analysis [126,127,128]. The utilisation of artificial intelligence facilitates the analysis of data collected from multiple sources, including sensors, cameras, and accident reports. This analysis enables the detection of patterns that may signify potential risks. The employment of machine learning algorithms has been demonstrated to enhance the efficacy of extracting valuable information from inspection data, facilitating the identification of defects associated with construction quality, and enabling the prediction of construction site accidents. The utilisation of artificial intelligence (AI) in the analysis of risk factors associated with various factors, including geographical location, the nature of the work, historical accident records, and prevailing weather conditions, facilitates enhanced safety management [129,130]. The utilisation of artificial intelligence in conducting hazard analyses has the potential to serve as a significant educational and training component. For instance, Uddin et al. [131] employed ChatGPT to identify threats. Furthermore, the utilisation of chatbots that are supported by artificial intelligence has the potential to serve as a highly effective medium for the purpose of raising awareness within the construction industry. Zhu et al. [132] demonstrated that the utilisation of a chatbot has the capacity to enhance the awareness of workers who are less experienced. The implementation of such a solution can offer a valuable educational component and can be incorporated into existing training programmes.

Recognising and monitoring the behaviour of workers on construction sites is crucial to managing quality workplace safety. Recent advances in computer vision technology suggest its potential to replace traditional manual supervision approaches [41]. The implementation of artificial intelligence has been demonstrated to facilitate the identification of appropriate behavioural patterns within the workforce, whilst concomitantly ensuring the protection of workers from hazardous incidents [133]. The field of artificial intelligence has also been applied to autonomous robotic systems and unmanned aerial vehicles with a view to enhancing safety in construction. Unmanned aerial vehicles equipped with artificial intelligence (AI) and thermal cameras have the capacity to perform inspections on large construction sites, thereby identifying potential hazards without the necessity of human involvement in high-risk work [134]. AI-supported UAVs have the capacity to detect irregularities in construction structures, such as cracks or other damage, which can prevent accidents or breakdowns. UAVs have the capacity to monitor areas that are challenging for humans to access, thereby facilitating enhanced safety oversight.

## 4. Evaluation of Modern Technologies for Improving Occupational Safety

### 4.1. General Characteristics and Future Research Directions

#### 4.1.1. Unmanned Aerial Vehicles and Inspection Robots

The utilisation of reality capture technology, encompassing unmanned aerial vehicles (UAVs) and inspection robots, signifies a substantial advancement in enhancing occupational safety. These technologies facilitate the comprehensive monitoring of working conditions, documenting the progression of tasks and identifying potential hazards without compromising human health and life.

The utilisation of reality capture technology has been demonstrated to enhance safety by minimising the presence of personnel in hazardous work zones. Additionally, it has been shown to improve the efficiency of inspections through the use of unmanned aerial vehicles and robots, which facilitate faster, more accurate, and repeatable measurements. This, in turn, enables a comparative analysis over time. The data obtained can be integrated with building information modelling (BIM) systems or geographic information systems (GIS) tools. These tools support a range of functions, including risk management, evacuation planning, the assessment of the technical condition of facilities, and the planning of corrective actions.

Despite the evident advantages, the implementation of these technologies in the context of work safety in the mining industry is constrained due to numerous limitations. As highlighted by Khan et al. [135] and Banerjee et al. [136], there are issues concerning the reliability and resilience of unmanned aerial vehicles in the event of failure, which give rise to concerns regarding their stable operation in the harsh conditions of mines. In addition, as noted by Garzia et al. [137], emotional reception and acceptance by employees are key but still poorly researched aspects that can have a significant impact on the effective integration of this technology with safety systems in mining. There is a paucity of empirical evidence on the long-term effects and actual adaptability of the systems considered by employees, indicating significant gaps in the current state of research.

Similarly, despite the evident safety benefits, the implementation of reality capture technology in construction faces a number of challenges. As indicated by Remenyte-Prescott et al. [138], Bassi [139], Heikkila et al. [140], Tan and He [141], and Xu et al. [142], reliability issues, regulatory ambiguities, and certification requirements have been identified as significant barriers to the widespread adoption of this technology. Furthermore, issues pertaining to data processing, accuracy, and real-time integration with existing security management systems persist as unresolved concerns. Moreover, there is a paucity of empirical evidence regarding the long-term effects and effectiveness of their implementation in complex building environments, thereby underscoring significant gaps in the current state of knowledge. It is recommended that future research endeavours concentrate on conducting exhaustive field assessments and conducting practical analyses. It is imperative to emphasise the necessity of conducting extensive testing under real-world construction conditions to reliably assess the adaptability of these systems. In the course of the research, consideration should be given to the integration of the technology into existing security management protocols and emergency response systems. A subsequent analysis should encompass user acceptance, training requirements, and operational best practices, all of which are instrumental in successful implementation and optimisation of security benefits.

In view of the dynamic evolution of reality capture, several avenues for future scientific research can be identified:

- It seems important to develop autonomous navigation and mapping systems in environments without a GPS signal, which will enable the effective use of robots in spaces without this signal;
- It is necessary to improve the methods of real-time spatial data analysis, including the implementation of artificial intelligence (AI) algorithms capable of automatic detection of threats and violations of security procedures;
- Research on the integration of different measurement platforms (e.g., UAVs, wheeled robots, and stationary devices) within a single safety management system, which will allow for a more holistic approach to monitoring and prevention;
- Research in the field of practical pilot implementations, allowing for a full assessment of the adaptability of UAVs and inspection robots in real mining conditions;
- Further research on user acceptance, psychological impact, and human–UAV/inspection robot interaction is warranted to ensure effective implementation and the maximisation of occupational safety benefits.

#### 4.1.2. Internet of Things and Sensors

A large number of accidents are documented in the construction and mining industries. Therefore, research should be conducted in both industries on the use of IoT-based technologies and sensors to improve occupational safety. Tabatabaee et al. [117] identified a lack of research on the use of the IoT in construction safety management. The integration of tools such as BIM and the use of machine learning in safety monitoring are set to become the future of the industry, with the aim of minimising risk and creating safer working environments on construction sites around the world [143,144].

The utilisation of sensors facilitates the uninterrupted aggregation of environmental data (e.g., dust, gas concentration, etc.), a process of paramount importance, particularly in environments characterised by a high degree of risk (e.g., environments with a risk of explosion or poisoning). The utilisation of IoT systems facilitates the real-time transmission of data to central management units, thereby enabling an immediate response when values exceeding permissible standards are detected [145,146]. The Internet of Things (IoT) is utilised for the purpose of monitoring the stability of both temporary and permanent structures, in addition to rock mass displacement, the movement of load-bearing elements, and weather conditions that have the potential to impact work safety. This enables continuous risk assessment and prevention of construction and mining disasters. Furthermore, sensors worn by workers enable the control of physiological parameters, location, and the identification of falls or lack of movement, which supports quick rescue operations [147]. Another aspect that should be considered is the enhancement of the management of the machine park and the implementation of predictive maintenance. The analysis of data from sensors installed on work equipment has been demonstrated to facilitate the prediction of equipment failures, the planning of technical inspections, and the avoidance of costly downtime [148,149]. Consequently, operational efficiency is enhanced, the risk of malfunctions during operation is reduced, and the exposure of employees to dangerous situations related to sudden equipment failure is minimised.

The Internet of Things, when interacting with industrial machinery and connected networks in mining operations, facilitates the real-time monitoring and control of production systems. This, in turn, leads to enhanced efficiency, reduced human intervention, and increased production. Furthermore, it contributes to enhanced occupational safety and reduced pollution. By leveraging advanced monitoring systems based on the IoT and deep learning, with the objective of continuously improving the ergonomics and functionality of traditional personal protective equipment (PPE), the goal is to minimise risk and create safer working conditions [150,151]. However, a holistic approach is imperative, integrating innovative technologies with a robust safety culture and employee awareness to ensure effective protection in this exacting work environment. Notwithstanding the numerous advantages, the implementation of IoT solutions also carries risks. Of these, the most significant are those pertaining to cybersecurity threats. The interception or manipulation of data transmitted via IoT systems has the potential to result in suboptimal operational decisions, which can have severe consequences in the context of construction and mining. The absence of adequate network security measures, the encryption of data transmission, and access authorisation has been demonstrated to increase vulnerability to hacker attacks [152,153]. A further significant issue pertains to the reliability of the sensors and communication systems themselves. In the event of sensor failure, erroneous measurements, or interruptions in data transmission, there is the potential for false alarms or a lack of warning in the event of a real emergency. In harsh environments, characterised by elevated levels of humidity, dust, or vibrations, the reliability of electronic equipment must be particularly high. This, in turn, generates additional costs related to the quality of components and their maintenance. The consideration of ethical and social issues pertaining to employee monitoring is of equal significance. The constant tracking of location and biological parameters may be perceived as an infringement on personal privacy, and the absence of transparent guidelines for data collection and processing can result in conflicts between employers and employees. In the domain of research concerning the utilisation of the IoT and sensors to enhance safety in construction and mining, the following aspects are of particular significance:

- Work on the development of autonomous decision-making systems, based on artificial intelligence and machine learning, in order to create systems capable not only of detecting threats but also of predicting their occurrence well in advance of time based on patterns of historical and current data;
- Research on improving the reliability and miniaturisation of sensors resistant to extreme environmental conditions;
- The development of solutions dedicated to industrial IoT, taking into account the specifics of critical infrastructure and ensuring protection of data integrity in real time;
- Interdisciplinary research covering social, legal, and organisational aspects of the implementation of new technologies—models of personal data management and transparent technology implementation policies that take into account the protection of employees’ privacy while increasing their security are necessary.

#### 4.1.3. Artificial Intelligence

The utilisation of artificial intelligence is becoming increasingly prevalent, and it has been demonstrated to possess significant advantages with regard to enhancing workplace safety. The integration of AI-based technologies, including image analysis, machine learning, predictive systems, and autonomous machines, holds considerable promise in enhancing the efficacy of safety management systems and contributing to a substantial reduction in accidents. The most significant aspect pertains to the capacity of AI systems to analyse extensive data sets in real time. In the fields of construction and mining, data from sensors, surveillance cameras, unmanned aerial vehicles, and devices worn by workers can be analysed via algorithms to identify hazards such as unsafe behaviour, machine failures, approaching hazardous zones, or deteriorating environmental conditions. Such systems have the capacity to generate automatic alerts, thereby significantly increasing the speed of response and reducing the risk of accidents [154]. The utilisation of artificial intelligence in data analysis has been demonstrated to facilitate the prediction of undesirable events and the prevention of equipment failures. This capability is enabled via the analysis of technical condition and operating data. The application of artificial intelligence has the potential to enhance the efficiency of work processes through automation. The deployment of autonomous robotic systems, machinery, or vehicles in particularly hazardous environments has the potential to mitigate human exposure to risk factors.

While the application of AI in the mining and construction industries holds considerable potential for enhancing workplace safety, it is important to acknowledge the numerous challenges associated with its implementation [155,156,157]. The efficacy of AI is contingent upon the calibre of the data and the dependability of the technical infrastructure, which can prove challenging to sustain within industrial contexts. A reliance on AI systems to the exclusion of other measures may result in a diminution of workers’ vigilance and a failure to adhere to conventional security procedures. Furthermore, the operation of numerous algorithms is characterised by a lack of transparency, which complicates the evaluation of their decision-making processes and the identification of their responsibilities in the event of an accident. It is also important to consider the significant privacy concerns that are raised by the use of AI to monitor employee health or behaviour. Inadequate management of this data has the potential to result in its misuse or discriminatory practices. The elevated expense of implementation is also a matter of concern, with the potential to intensify the technological disparities between large and small companies. Furthermore, the automation of certain processes has the potential to result in the downsizing and dehumanisation of the work environment.

In view of the dynamic development of technology, further research should focus on several key areas:

- Developing AI algorithms to better understand and verify the decisions made by AI systems, which will increase their trust and enable more effective crisis management;
- Conducting interdisciplinary research on the integration of AI with work ergonomics, psychology and social engineering, which will enable the creation of more user-friendly systems supporting safety;
- Developing standards and legal norms for accountability for decisions made via autonomous systems, as well as defining a framework for auditing and certifying AI-based solutions in high-risk environments.

Artificial intelligence has been identified as a potentially effective tool for enhancing safety in the construction and mining industries, with the potential to deliver a range of innovative solutions. However, it should be noted that, in the current context, AI should be regarded as a tool that supports, rather than replaces, human capabilities. The implementation of this system must be conducted in a manner that is both responsible and transparent, in accordance with the principles of ethics and occupational safety.

### 4.2. Inter-Technological Integration—Opportunities and Limitations

Modern technological developments are opening up new opportunities for enhancing occupational health and safety. The integration of advanced solutions is pivotal in driving the transformation of methodologies employed in the domains of occupational safety management within the sectors of mining and construction. Utilised individually or in synergy, these technologies facilitate enhanced monitoring and analysis of workplace hazards, as well as the proactive prediction and prevention of accidents and hazardous incidents. Despite the numerous benefits, the integration and simultaneous application of these solutions also present significant barriers. The potential for combining them, as well as the limitations of such combinations, are summarised in Table 2.

#### 4.2.1. Opportunities for the Use and Mixing of Modern Technologies

The integration of unmanned aerial vehicles, inspection robots, the Internet of Things, and sensors is imperative to enhance work safety. In recent years, there has been a notable increase in the utilisation of contemporary technologies, such as autonomous robots, within industrial settings. This development has enabled enhanced efficiency and precision in the monitoring of risks and the identification of potential hazards [158]. The utilisation of UAVs and robotic systems in inspection tasks is of paramount importance, as they possess the capacity to access inaccessible locations and operate in challenging environments. These systems facilitate the capacity of emergency services and enterprises to detect variations in terrain and evaluate the condition of both objects and the environment. This, in turn, has a substantial impact on decisions pertaining to work safety [159,160,161]. Moreover, the integration of unmanned aerial vehicles and inspection robots with the IoT and sensors has resulted in the development of intelligent monitoring systems that combine data from multiple sources and analyse it in real time. This facilitates the expeditious identification of threats by enforcement agencies, enabling immediate mitigation or elimination [162]. In the context of mining projects, for instance, sensors have been employed to monitor air conditions, a crucial aspect of preventing accidents involving gas explosions [163]. In the field of construction, the integration of these technologies facilitates the incorporation of 3D scanning systems to assess construction progress and monitor any deviations from the construction plan. Moreover, the integration of these technologies facilitates the continuous identification of problems such as structural damage or installation errors, thereby contributing to the minimisation of the risk of accidents [164]. Another example is the utilisation of UAVs equipped with sensors that collect data on landslide phenomena. The integration of this data with data analysis has been demonstrated to result in substantial improvements in work safety [165].

The utilisation of artificial intelligence is becoming increasingly prevalent. The integration of artificial intelligence with unmanned aerial vehicles or inspection robots to enhance work safety is anticipated to garner increased acceptance and validation. The initial domains of application for AI and UAVs pertain to the monitoring and evaluation of working conditions within the construction industry and the mining sector. Examples of such systems include the inspection of building structures, the detection of defects in building materials, and the monitoring of weather conditions that may affect work safety [44,45,166]. The analytical capabilities of AI enable such systems to function in two distinct yet interconnected ways. Firstly, they are able to identify problems. Secondly, they can predict the development of such problems and suggest risk management strategies. In the context of mining operations, the deployment of unmanned aerial vehicles (UAVs) or inspection robots has been proposed as a means of transmitting location information in the event of a hazardous incident. This approach is predicated on the premise that it would facilitate the expeditious evacuation of personnel [167,168]. Furthermore, the integration of AI within autonomous inspection robots serves to augment their operational capabilities. Robots have been developed for the purpose of conducting inspections in locations that are inaccessible to humans, such as confined spaces or hazardous areas where the risk to health and life is particularly high. This approach has been shown to eliminate many hazards, such as exposing people to dangerous situations.

The Internet of Things encompasses a variety of devices, including wearable technologies and sensors, which facilitate uninterrupted observation of working conditions and environmental parameters. A fatigue detection system is an example of such a system. This system uses sensors to analyse biological data, thereby enabling the rapid recognition of signs of fatigue and the triggering of an alarm to prevent accidents. In a similar manner, sensors affixed to vehicles are capable of monitoring driver behaviour and the technical condition of the vehicle, thereby ensuring its safe operation. The synergy between these devices and AI algorithms has resulted in the development of advanced early warning systems that identify potential hazards based on pattern analysis in data, allowing for timely intervention [169]. In the field of mining, where personnel frequently operate in arduous conditions, the significance of AI-augmented sensors in ensuring occupational health and safety becomes paramount.

The integration of UAVs and inspection robots with virtual reality and augmented reality technology, exoskeletons, and contemporary personal and collective protective equipment has proven to be a successful approach. The utilisation of AR facilitates the real-time visualisation of intricate processes, while concurrently providing information regarding prevailing hazards through image transmission from unmanned aerial vehicles or inspection robots. In a similar vein, the utilisation of unmanned aerial vehicles or inspection robots equipped with exoskeletons facilitates the management of hazardous and emergency scenarios. The integration of unmanned aerial vehicles or inspection robots with contemporary personal or collective protective equipment will facilitate real-time risk monitoring and evaluation.

The integration of the Internet of Things and sensors with virtual reality and augmented reality technology, exoskeletons and innovative personal or collective protective equipment offers significant opportunities to improve work safety. However, in order to achieve these benefits, it is necessary to adopt comprehensive strategies that take into account both the technical and human aspects of introducing new technologies. The utilisation of data collected from the IoT and sensors will facilitate the more effective design of training scenarios in a VR environment. The analysis of this data will facilitate the identification of training areas of particular importance. Furthermore, the integration of sensor data will facilitate enhanced visualisation of processes within AR. The combination of the IoT and sensors with exoskeletons is of particular importance in the context of ergonomics. The integration of these components will facilitate the analysis of biomechanical data, health and fatigue monitoring during the utilisation of exoskeletons, while concurrently providing physical and ergonomic support. The integration of sensors with personal and collective protective equipment will enable real-time monitoring and analysis of the working environment. The IoT technology has the capacity to facilitate the continuous monitoring of employees’ health. For instance, sensors can be utilised to measure employees’ vital signs, including body temperature, oxygen saturation, and other physiological indicators [170]. The results of these measurements can be tracked in real time and used to respond quickly to hazards, which in turn reduces the risk of serious incidents in the workplace.

The development of solutions to support occupational safety is dependent on the interoperability of artificial intelligence and augmented and virtual reality, exoskeletons and modern personal or collective protective equipment. The integration of AI with VR facilitates the simulation of technological processes, thereby enabling the analysis of risks associated with specific activities during the implementation of a training scenario. The implementation of preventive measures can be achieved through the observation and analysis of user behaviour. In the context of exoskeleton applications, the integration of AI has the potential to enhance the functionality of these devices by facilitating more ergonomic interaction with the user and optimising movement mechanics. For instance, exoskeletons can be integrated with AI systems that continuously monitor the user’s posture and load, adjusting their range of motion to the individual needs and limitations of the employee. Such solutions have been shown to have the potential to significantly reduce the risk of strain injuries. In the context of innovative protective measures, integration with IS systems, as well as with sensors, has the potential to automate work condition monitoring processes. For instance, the integration of the IoT with sensors, in conjunction with AI, facilitates the real-time monitoring of the working environment. In the event of dangerous conditions being detected, these systems have the capacity to automatically notify employees of the hazard and control evacuation procedures.

To confirm the results obtained, three empirical case studies were cited, illustrating the successful integration of technology in the construction, oil, and mining sectors. In Chennai, a system was developed in which a drone equipped with a 4K camera and the Faster-R-CNN model identified workers without safety helmets with 93.1% accuracy at 27 fps, enabling immediate response by health and safety services [171]. In the Rub-al-Chali desert, the use of a UAV platform with a CNN network and the Dynamically Constrained Accumulative Membership Fuzzy Logic algorithm increased the effectiveness of pipeline crack detection, reducing the inspection of a 40-kilometre section from three days to five hours [172]. In turn, in the UNEX-1000 test mine, the cooperation of ANYmal C robots, a tracked UGV and micro-drones, sharing a network of gas sensors and LiDAR-SLAM, reduced the number of human entries into hazardous areas by 42% and shortened the inspection time of workings by 30% over a six-month period [167].

#### 4.2.2. Limitations on the Use and Mixing of Modern Technologies

Despite the numerous advantages of integrating technological solutions to enhance workplace safety, there are also substantial limitations. The challenges associated with the human–machine interface and data security are of paramount importance. Research has demonstrated that a lack of acceptance of technology among employees, coupled with concerns regarding privacy, can impede the adoption of contemporary systems [173]. Moreover, it is imperative to acknowledge that, in numerous instances, the financial viability of investments in contemporary technologies is constrained by protracted return on investment cycles and an absence of comprehensive comprehension regarding the advantages inherent in such solutions.

The quality of integration of technologies, and consequently safety, may be affected by environmental adaptation challenges. Such challenges may include dust, vibrations, humidity, temperature (stable but significantly elevated/lowered temperature or large daily temperature amplitude), shocks (natural or caused by heavy machinery) and communication availability (a lack of or interference with GPS and a lack of or interference with radio signals). Mining will be subject to a range of environmental factors that are distinct from those relevant to construction. Consequently, technological adaptation must be closely linked to these factors and industry needs. In the context of mining operations, the most significant challenges encountered by professionals include elevated temperatures, humidity, dust, vibrations, natural shocks, and inadequate communication. It is important to note that these factors vary between underground and opencast mining. Conversely, in the field of construction, the most significant challenges pertain to the variability of weather conditions, such as strong winds and fluctuating temperatures.

The inherent complexity of systems has been demonstrated to have a substantial impact on the reliability of the technologies utilised. This is especially the case in challenging conditions, where equipment must be durable and adapted to harsh working environments. The occurrence of system failures has the potential to engender novel hazards. Furthermore, technological advancements frequently exceed the pace of regulatory and safety standards, impeding their comprehensive implementation and certification.

In the contemporary context of the pervasive utilisation of data-driven systems, data security has emerged as a pivotal concern. Systems that collect data from multiple sources are vulnerable to cyber attacks, which can have serious consequences. Consequently, the development of effective strategies to protect systems against potential threats is imperative.

Technological advances have engendered new opportunities for the protection of health and life. Concerns pertaining to data privacy (i.e., legal and ethical issues) have the potential to engender apprehensions with regard to information security, particularly in relation to the collection and analysis of user information. The intensified monitoring of employees gives rise to a number of legal and ethical issues, the neglect of which has the potential to erode staff trust and engender legal risks for companies. Consequently, when implementing innovative technologies, employers must consider the ethical and privacy implications of monitoring their employees (e.g., tracking physiological parameters). It is imperative that these issues are regulated in accordance with the prevailing legislation whilst ensuring the protection of the interests of both employees and employers.

Another important aspect is the need for training for employees to ensure an adequate level of skills in the use of modern tools. Research has indicated that a paucity of adequate training can engender concerns and uncertainty, which can have a detrimental effect on the utilisation of technology in practice [174]. Furthermore, it is important that these technologies are designed with ease of use in mind to minimise stress associated with new tools and increase employee engagement [175].

Despite the measurable successes of integrating modern technologies, the literature also notes significant failures. An analysis of 54 autonomous flights revealed that GPS spoofing increased the average positioning error to 20.45 m, and jamming reduced the percentage of completed missions by 40%, with even the best support vector machine classifier achieving only 56% recall for spoofed signals, highlighting the scale of cyber risk [176]. A classic experiment conducted in Texas showed that a simple spoofing transmitter can completely take control of a rotor drone and cause it to crash, emphasising the criticality of navigational redundancy [177]. In construction, however, the main barriers remain organisational factors, where a systematic review of UAV implementations has shown that privacy concerns, a shortage of trained operators, and ambiguous data management procedures significantly limit the scalability of even technically successful projects [178].

#### 4.2.3. Practical Ways to Reduce the Risks Facing the Use and Mixing of Modern Technologies

Case studies show that the combined use of UAVs, robots, IoT, and AI reduces staff exposure to hazards and shortens inspections by 30–40%. The prerequisites for success are sensor redundancy and training models on data from local conditions, as well as the close integration of monitoring systems with health and safety procedures and data warehouses, which ensures a rapid response from services. Failures are mainly due to cybersecurity gaps (GPS spoofing and jamming) and extreme environmental conditions that reduce equipment reliability. In addition, organisational factors determine the scale of implementation: user acceptance, operational competence, and transparent privacy policies. Effective implementation requires participatory design, a comprehensive training programme, RF interference resistance testing, and emergency procedures based on inertial-visual odometry. Only by linking real-time data streams with planning systems (enterprise resource planning) and robust cybersecurity and data management frameworks can the full potential of these technologies be realised.

In practice, mitigating the risks associated with the integration of modern technologies boils down to a few basic steps. Firstly, IoT devices should only transmit data in encrypted tunnels (TLS/DTLS 1.3) and receive signed, verified firmware updates in accordance with the SUIT architecture, which makes it much more difficult to take control or inject malicious code [179]. Second, artificial intelligence systems should be covered by a formal management system, e.g., according to ISO 12164-4, and regularly audited for bias and model drift to ensure that results remain reliable and fair [180]. Thirdly, BSP platforms and mobile robots should be protected against spoofing and jamming by combining GNSS signals with inertial and vision navigation and securing the control link with AES-GCM encryption and frequency hopping, and in the event of loss of orientation, the device should switch to a safe ‘fail-soft’ mode [181]. Fourthly, collaborative robots and exoskeletons must be equipped with an immediate stop function and force monitoring in accordance with ISO 10218-1 and ISO/TS 15066, which minimises the risk of injury to the operator [181,182]. Finally, each of these technologies must be supported by clear procedures, regular penetration tests, and staff training. Only such a comprehensive approach translates into a real increase in safety while maintaining flexibility and the scale of implementation.

## 5. Conclusions

The modern approach to occupational safety in high-risk sectors, such as mining and construction, is increasingly based on the integration of modern technologies. Unmanned aerial vehicles, inspection robots, the Internet of Things, smart sensors, and artificial intelligence are significantly influencing the new quality of safety management in the workplace. These solutions offer capabilities that far exceed those of traditional methods of hazard and dangerous event prevention and response. The utilisation of advanced technologies facilitates a transition from a reactive to a predictive model, in which hazards are identified and eliminated before an undesirable situation occurs. A fundamental element of this process is the capacity to collect, process, and analyse data in real time. Information from distributed systems monitoring working conditions and the performance of professional activities is integrated and interpreted using machine learning algorithms, which support operational decisions focused on occupational safety and significantly reduce the risk of accidents.

The long-term development prospects for technology indicate a growing role for digital systems and their integration into intelligent safety systems. The increased availability of technologies, along with their continuous improvement and expansion of technical capabilities, will facilitate their wider implementation, thereby significantly enhancing safety in the workplace. The advent of technologies that facilitate occupational safety in the mining and construction industries is indicative of the contemporary challenges faced by industry. It is anticipated that, in the forthcoming years, there will be a progressive augmentation in the establishment of novel technologies and the integration of contemporary systems to bolster the management of occupational safety.

The analyses conducted in this article suggest that the integration of at least two technologies—for example, combining unmanned aerial vehicles with AI algorithms—can lead to a statistically significant reduction in accident rates, as it enables earlier detection of hazards. However, as the digital ecosystem expands, the surface area for potential attacks grows, rendering the design and implementation of uniform cybersecurity standards imperative. It is equally important to consider user acceptance of modern equipment, since this will ultimately determine the actual use of technology in everyday practice.

For a more comprehensive assessment of the long-term success of implementations, conclusions should be extended to include socio-cultural dimensions: the acceptance of new technologies depends on variables such as fear of job loss, trust in algorithms, the generational ‘digital divide’, and the maturity of the plant’s safety culture. A socio-technical implementation framework based on participatory co-design involving employees and trade unions, the transparent communication of benefits, and clear data protection rules is recommended. A parallel ‘skills & trust’ programme combining on-the-job training, e-learning, and VR/AR simulations is necessary, the effectiveness of which is measured, for example, using the declared level of trust or response time in test scenarios. During the operational phase, regular surveys and focus groups should be conducted, ‘adoption dashboards’ (system usage, incidents, and bias audit results) should be published, and the results should be linked to reskilling programmes and incentive policies. Such a technology-integrated approach creates a solid foundation for proactive and widely accepted occupational safety management.

It is recommended that future research concentrate on the advancement of current technologies and the identification of novel technological solutions to enhance work safety, the integration of visual and sensory data across multiple domains, the establishment of reliable indicators to measure the return on investment in safety, and the long-term health implications associated with the utilisation of operator support devices. The dynamic development of innovation in mining and construction is a response to two key factors. Firstly, the growing complexity of processes, and secondly, the pressure to achieve zero accidents. All indications are that, in the coming years, we will see further intensive research and development and the implementation of systems that will enable increasingly proactive, integrated, and effective occupational safety management.

## Figures and Tables

**Table 1:** Keywords and search results (own study).

Technology	Keywords			
	Mining Engineering	Results	Civil Engineering	Results
Unmanned aerial vehicles (UAV) and inspection robots	drone OR UAV OR robotic inspection AND mining engineering OR mining AND safety	45	drone OR UAV OR robotic inspection AND civil engineering OR construction industry AND safety	34
Internet of Things (IoT) and sensors	IoT OR Internet of Things OR sensors AND occupational safety AND mining OR mining engineering	68	IoT OR Internet of Things OR sensors AND occupational safety AND civil engineering OR construction industry	33
Artificial intelligence (AI)	AI OR artificial intelligence AND occupational safety AND mining OR mining engineering	13	AI OR artificial intelligence AND occupational safety AND civil engineering OR construction industry	16

**Table 2:** Analysis of the possibilities of combining modern technologies in mining and construction—opportunities and limitations (own study).

	Unmanned Aerial Vehicles and Inspection Robots	IoT and Sensors	Artificial Intelligence	Augmented Reality (AR) and Virtual Reality (VR)	Innovative Personal Protective Equipment (PPE) and Collective Protective Equipment (CPE)	Exoskeletons
	Opportunities					
Exoskeletons	Management of hazardous and emergency situations	Biomechanical data analysis; Health and fatigue monitoring; Physical and ergonomic support	Assist with high-risk physical work; Motor support; Managing fatigue and interruptions	Haptic feedback; Synchronisation of motion data	Motion support and increased worker safety	
Innovative personal protective equipment (PPE) and collective protective equipment (CPE)	Real-time monitoring and risk assessment	Monitor and analyse the work environment in real time; Manage the operation and condition of protective equipment; Responding to hazardous and accident situations; Data analysis and prediction of hazards and accidents	Creating smart PPE and CPE; Automation of collective protection system	Compatibility of purpose with actual activities performed; Test simulations of PPE and CPE.		Lack of standards and norms; Compatibility problems with security systems
Augmented reality (AR) and virtual reality (VR)	Creating realistic 3D environments; Reconstructing accidents; Monitoring working conditions and identifying hazards; Use of real-time AR in work environments	Integrating data from IoT devices into a VR environment; Industrial training and simulation	Machine learning for VR environment adaptation; Personalisation of VR/AR scenarios; Analysis of user response behaviour of VR/AR technologies; Risk assessment		A false sense of security; Ergonomic and health problems	Cybersecurity threats; Technical errors and physical consequences
Artificial intelligence	Monitoring working conditions and identifying hazards; Predicting accidents and failures; Mapping and 3D modelling of the work environment	Real-time monitoring and analysis of the work environment; Predicting accidents and failures; Access control and employee identification		Algorithmic errors; Improper data analysis; Data leakage; Invasion of privacy; Qualified personnel requirement	Algorithmic errors; Improper data analysis; Data leakage; Invasion of privacy; Cybersecurity threat	Algorithmic errors and improper data analysis; Data leakage; Invasion of privacy; Cybersecurity threat; The complexity of technology integration
IoT and sensors	Real-time monitoring of the work environment; Remote inspection and control of hazardous sites; Data analysis and prediction of hazards and accidents		Reducing employee vigilance; AI misinterpretation of data; Data leakage; Invasion of privacy; Cybersecurity threat; Ethical and legal issues	Data leakage; Invasion of privacy; Cybersecurity threat	Unreliability of technology; Data leakage; Invasion of privacy; Cybersecurity threat; Need for qualified personnel	A false sense of security; Unreliability of technology; Cybersecurity threat; The complexity of technology integration
Unmanned aerial vehicles and inspection robots		Data leakage; Invasion of privacy; Cybersecurity threat; The complexity of technology integration; Unreliability of technology; Dependencies on communication systems	Algorithmic errors; Improper data analysis; Data leakage; Invasion of privacy; Cybersecurity threat; The complexity of technology integration	A false sense of security; Technical issues and hardware compatibility; Cybersecurity threat; Data leakage; Invasion of privacy; Need for qualified personnel	A false sense of security; Compatibility issues; Cybersecurity threat	Lack of standards and norms; Threats to cybersecurity; Complexity of technology integration
	Limitations					

## Data Availability

The original contributions presented in this study are included in the article. Further inquiries can be directed to the corresponding author.

## Abbreviations

The following abbreviations are used in this manuscript:

VR	Virtual reality
OSH	Occupational safety and health
UAVs	Unmanned aerial vehicles
IR	Inspection robots
IoT	Internet of Things
AI	Artificial intelligence
CSIR	The Council for Scientific and Industrial Research
CMI	The Center for Mining Innovation
AR	Augmented reality
BIM	Building information modelling
OSHA	Occupational Safety and Health Administration
PAD	Personal alarm device
NIOSH	The National Institute for Occupational Safety and Health
SMAC	Smart monitoring and control
SMRD	Spokane Mining Research Division
WSN	Wireless sensor network
AMS	Air-monitoring system
LED	Light-emitting diode
UWB	Ultra-wide band
GIS	Geographic information system
FEM	Finite element method
PPE	Personal protective equipment
CPE	Collective protective equipment
